# Supplementary material for: Cost-Effectiveness Analysis of the Prophylactic Use of Ertapenem for the Prevention of Surgical Site Infections after Elective Colorectal Surgery
Source: Antibiotics (Basel). 2021 Mar 4;10(3):259. doi: 10.3390/antibiotics10030259 (PMC7999678; doi:10.3390/antibiotics10030259)
Supplement: Supplementary file 1 [file antibiotics-10-00259-s001.zip › Supplementary File 1 - Systematic review.docx]

**Systematic review – additional information.**

**Methods**

A systematic review was conducted independently by two Authors (C.V. and A.C.). PubMed, Embase and Cochrane databases were searched for studies measuring the efficacy of ertapenem in preventing SSIs following colorectal procedures. Randomized controlled trials, case-control and cohort studies, as well as systematic reviews published through April 30^th^, 2020 were included. No restrictions on language or year of publication were imposed. Studies were considered eligible if they reported extractable data on two outcomes: the proportion of SSIs and/or on the proportion of AMR pathogens isolated from SSIs. Data from all ertapenem study arms were extracted. Authors of included studies were contacted if information on the outcomes of interest was not specified in the published articles.

1. Search terms

**Pubmed**

(("Colorectal Surgery"[Mesh] OR (("Colon"[Mesh] OR "Rectum"[Mesh] OR colon OR colonic* OR rectum* OR rectal* OR colorect* OR colo-rect*) AND ("surgery" [Subheading] OR surger* OR surgi*)) OR colectomy) AND ("Ertapenem"[Mesh] OR Ertapenem OR Ertapenem-Sodium OR "Administration, Intravenous"[Mesh] OR intravenous) AND ("Antibiotic Prophylaxis"[Mesh] OR prophylaxis OR antibiotic prophylaxis OR antimicrobial prophylaxis) AND ("Surgical Wound Infection"[Mesh] OR infection*))

**Embase**

(('colorectal surgery'/exp OR 'colon resection'/exp OR 'colon resection' OR (('colon'/exp OR 'rectum'/exp OR colon OR colonic* OR rectum* OR rectal* OR colorect* OR 'colo rect*') AND ('surgery'/exp OR surger* OR surgi*))) AND ('ertapenem'/exp OR 'ertapenem sodium' OR ertapenem OR 'intravenous drug administration' OR intravenous) AND ('antibiotic prophylaxis'/exp OR prophylax* OR 'antimicrobial prophylaxis' OR 'antibiotic prophylaxis') AND ('surgical infection'/exp OR infection*))

**Cochrane**

MeSH descriptors: [Colon], [Rectum], [General Surgery], [Ertapenem], [Antibiotic Prophylaxis], [Administration, Intravenous]

Other included search terms: colon OR colonic* OR rectum* OR rectal* OR colorect* OR colo-rect*, surger* OR surgi*, Ertapenem OR Ertapenem-Sodium, antibiotic prophylaxis OR antimicrobial prophylaxis OR prophylaxis

1. Data extraction

**Primary outcome**: proportion of SSIs in colorectal procedures for which ertapenem was administered as the only surgical prophylaxis.

- Denominator: number of participants in ertapenem arms minus participants whose outcomes were missing,
- Numerator: number of participants with postoperative infections.

**Secondary outcome**: proportion of AMR isolates from SSIs following colorectal procedures for which ertapenem was administered as the only surgical prophylaxis.

- Denominator: number of isolated micro-organisms,
- Numerator: number of isolates resistant to ertapenem.

**Results**

**Suppl. Figure 1**. Inclusion and exclusion diagram.


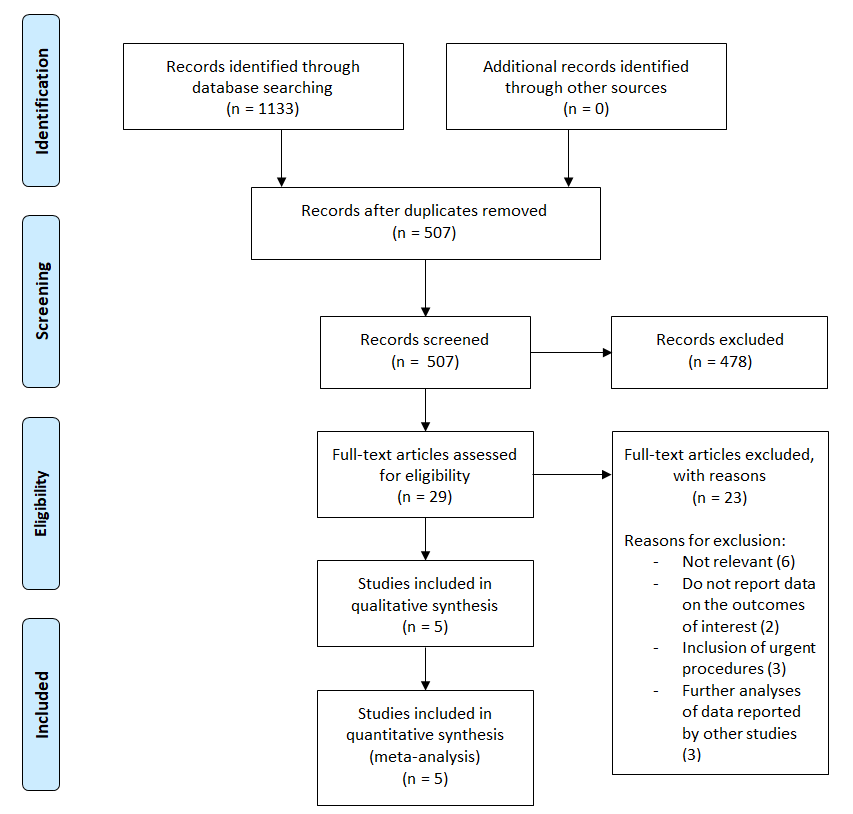


**Suppl. Table 1**. Characteristics of included studies.

| Reference | Study type | Location | Study period | Population (surgical procedure) | Exclusion criteria | Follow-up | Ertapenem timing, dosage and duration | Notes |
| --- | --- | --- | --- | --- | --- | --- | --- | --- |
| Leng et al, 2014 | Randomized controlled double-blind study | China (multicentric: 25 sites) | December 2010 – December 2011 | 526 adult patients undergoing elective colon or colorectal surgery by laparotomy | Patients with emergency procedures, second surgery anticipated within 4 weeks, scheduled laparoscopy-assisted surgery, isolated rectal procedure, revision of a previous operation involving large bowel resection, active inflammatory bowel disease involving the colon, bacterial infection requiring systemic antimicrobial therapy within 1 week prior to surgery, contraindication to the study drugs, immunosuppression, neutropenia at screening, pregnant or nursing women, considered unlikely to complete the study based on the investigator’s judgement. | 4 weeks | Single 1g dose within 2 hours prior to surgical incision, administered as a 30 min infusion. | Industry sponsored; study design, data collection and analysis by Merck&co., Inc. |
| Deierhoi et al, 2013 | Retrospective cohort study | USA (multicentric: 112 hospitals) | 2005 - 2009 | 5750 elective colorectal operations | Missing prophylactic antibiotic agent information or use of a non SCIP (Surgical Care Improvement Project) -approved antibiotic. | 30 days | Not specified. | Both LPS and open procedures were included.  Organ space SSI were not included in the outcomes. |
| Itani et al, 2006 | Randomized controlled double-blind study | USA (multicentric: 51 centres) | May 2002 – March 2005 | 672 adult patients undergoing elective open surgery of the colon or rectum with sufficient time for bowel preparation | Patients with emergency procedures, second planned surgery requiring antibiotic prophylaxis, elective procedure for revision of a previous surgery, laparoscopy-assisted surgery, isolated rectal procedure, active inflammatory bowel disease involving the colon, bacterial infection requiring systemic antimicrobial therapy up to 1 week prior to surgery, contraindication to the study drugs, immunosuppression, neutropenia, aminotransferase levels or prothrombin times at least three times the upper limit of the normal range, pregnant or nursing women. | 4 weeks | Single 1g dose infused over a 30-minute period within 60 minutes prior to surgical incision. | Industry sponsored; study design, data collection and analysis by Merck&co., Inc. |
| Kuriakose et al, 2019* | Retrospective cohort study | Michigan (multicentric: 70 hospitals) | January 2013 – February 2018 | 9949 adult patients undergoing elective colectomy and receiving SCIP -recommended antibiotics | Patients with no recorded antibiotic information, no 30-day follow up or with additional antibiotics. | 30 days | Not specified. | Both LPS and open procedures were included. |
| Nutman et al, 2019 | Prospective non-randomized, non-blinded interventional study | Israel, Switzerland and Serbia (multicentric: 3 hospitals) | 2012-2017 | 478 adult patients undergoing elective colorectal surgery. | Patients with hypersensitivity or other contraindication to the study drugs, that could not be followed for 30 days post-surgery, or had a documented infection at the time of surgery that necessitated antibiotic treatment. | 30 days | Single dose of IV ertapenem 1g up to 1 hour before surgery. | Screening for ESBL-PE carriage and personalized prophylaxis: ESBL-PE carriers received ertapenem. |

*Authors which provided further information upon request.

**Suppl. Table 2**. Results of included studies.

| Reference | Outcome 1 – Sample size, n of patients receiving ertapenem | Outcome 1 – n of SSIs in patients receiving ertapenem | Outcome 2 – Sample size, n of micro-organisms isolated and tested for resistance (ertapenem group) | Outcome 2 – Resistant isolates (ertapenem group) |
| --- | --- | --- | --- | --- |
| Leng et al, 2014 | 251 | 5 | No data on susceptibility testing. | No data on susceptibility testing. |
| Deierhoi et al, 2013 | 249 | 36 | No culture data. | No culture data. |
| Itani et al, 2006 | 338 | 62 | 92 | 1/11 tested |
| Kuriakose et al, 2019 | 2117 | 143* | No culture data. | No culture data. |
| Nutman et al, 2019 | 221 | 35 | No data on carbapenem resistance. | No data on carbapenem resistance. |

*Information provided upon request.

**References**

Deierhoi RJ, Dawes LG, Vick C *et al*. Choice of intravenous antibiotic prophylaxis for colorectal surgery does matter. *J Am Coll Surg* 2013; 217: 763–9.

Itani KMF, Wilson SE, Awad SS *et al*. Ertapenem versus cefotetan prophylaxis in elective colorectal surgery. *N Engl J Med* 2006; 355: 2640–51.

Kuriakose JP, Vu J, Karmakar M, *et al.* β-Lactam vs Non-β-Lactam Antibiotics and Surgical Site Infection in Colectomy Patients. *J Am Coll Surg* 2019; 229: 487-496.e2.

Leng XS, Zhao YJ, Qiu HZ, *et al.* Ertapenem prophylaxis of surgical site infections in elective colorectal surgery in China: A multicentre, randomized, double-blind, active-controlled study. *J Antimicrob Chemother* 2014; 69: 3379–86.

Nutman A, Temkin E, Harbarth S, *et al.* Personalized Ertapenem Prophylaxis for Carriers of Extended-spectrum β-Lactamase-producing Enterobacteriaceae Undergoing Colorectal Surgery. *Clin Infect Dis* 2020; 70: 1891–7.
